# Improvisation is a novel tool to study musicality

**DOI:** 10.1038/s41598-022-15312-5

**Published:** 2022-07-22

**Authors:** Michael W. Weiss, Isabelle Peretz

**Affiliations:** International Laboratory for Brain, Music, and Sound Research (BRAMS), Department of Psychology, University of Montreal, Montreal, QC Canada

**Keywords:** Psychology, Human behaviour

## Abstract

Humans spontaneously invent songs from an early age. Here, we exploit this natural inclination to probe implicit musical knowledge in 33 untrained and poor singers (amusia). Each sang 28 long improvisations as a response to a verbal prompt or a continuation of a melodic stem. To assess the extent to which each improvisation reflects tonality, which has been proposed to be a core organizational principle of musicality and which is present within most music traditions, we developed a new algorithm that compares a sung excerpt to a probability density function representing the tonal hierarchy of Western music. The results show signatures of tonality in both nonmusicians and individuals with congenital amusia, who have notorious difficulty performing musical tasks that require explicit responses and memory. The findings are a proof of concept that improvisation can serve as a novel, even enjoyable method for systematically measuring hidden aspects of musicality across the spectrum of musical ability.

Music invented in the moment of performance, termed *improvisation*, can be regarded as a honed skill reserved for expert musicians. Yet there are indications that improvisation is a natural, widespread behavior. Improvisation is common in many of the world’s musical traditions^1^, young children sing novel songs or rework familiar songs spontaneously^2^, and the vast majority of adults report improvisational singing in the absence of other people and music (74%; see Supplementary Fig. S1). Sung improvisation in particular does not require instrumental expertise^3,4^, making it accessible to non-musician participants, who constitute most of the population. Thus, improvisation represents a unique chance to uncover the rules acquired by the general population in everyday music—for example, the use of a limited number of discrete pitches, or integer ratios between tone onsets—just as speech does for language.

Musicality can be defined as, “a natural, spontaneously developing set of traits based on and constrained by our cognitive abilities and their underlying biology”^5^. Here, we examine whether sung improvisations demonstrate what has been proposed as one of the fundamental components of musicality: tonal organization of pitch^6,7^. Most tonal music uses 4–7 focal pitches, forming a scale. In such music, scale tones are organized around a central tone, sometimes called the tonic, which usually starts and ends a musical piece. Among the other scale tones, there is a hierarchy of importance or stability^8^. Nonscale tones are the least stable and often sound “sour”. These tonal principles allow, for example, any individual to detect an out-of-scale note in the musical surface. The organizational principle of tonality is largely absent in speech^9,10^, yet the presence of discrete pitches, often forming nonequidistant scales, is ‘statistically universal’ across musical systems^11^, even as scales differ across societies^12^. Tonal hierarchical organization of pitch is central because it facilitates perception, memory, and performance by creating expectancies^13,14^. We define tonality as adherence to a scale, specifically the major and minor scales of Western tonal music, which are most likely to be culturally familiar to our Canadian participants, as well as the tendency to return to the tonic at the end of a melody.

Prior behavioral studies of inner tonal knowledge have used paradigms that induce a sense of tonality^15^. For example, the classic probe tone technique consists of presenting a tonal sequence of pitches ending on a variable probe tone. Listeners rate whether the final tone fits the preceding context^16^. In Western listeners, the ratings typically follow a profile where the tonic and closely related tones (the fifth and third degrees) are preferred over other scale and out-of-scale tones. Another widely used method consists of presenting a priming context of notes or chords that generate tonal expectancies for the final target event. An irrelevant change on the target event, such as a change of timbre, will be more rapidly detected if the chord conforms to tonal expectations^17^. Variations of these methods reveal implicit tonal knowledge among children as young as 4–5 years old, nonmusicians, and even amusic individuals who have little or no awareness of music rules or theory^18–22^. Production paradigms (*‘sing the next note’*) are less common and tend to be restricted to musically experienced participants^23^. In all cases, however, a musical context guides the behavioral response.

Tonal induction created by the presentation of a musical context is akin to presenting an incomplete sentence and asking the participant to choose or produce only the final word, whereas one could ask the participant to invent the sentence themselves. The advantage of freeform production, as in sung improvisation, is that participants may directly express an abstracted rule (e.g., scale) in an unconstrained context, whereas tonal knowledge measured by tonal induction may simply reflect an accumulation of heard pitch frequencies. Indeed, there is a debate over the representation of implicit knowledge of music and whether it truly involves abstract rules or is simply the accumulation of knowledge fragments^24^. Differentiating between these possibilities requires testing individuals who lack metacognitive awareness of musical rules, and who are not guided by the paradigm.

Individuals with congenital amusia are an ideal population to test for the presence of tonal knowledge without awareness. These rare cases, estimated to comprise 1.5–4% of the population^25^, demonstrate a lifelong inability to detect out-of-scale tones in melodies that typically elicit strong tonal expectations^26^. Yet, these out-of-scale tones elicit similar electrical responses in the typical and amusic brain^27,28^. Such indirect measures indicate that some tonal knowledge is spared^21,27^ and could serve as a basis for interventions. However, the notion of tonal knowledge without awareness rests on the method of tonal induction and may not reflect the ability to follow the rules of tonality without musical context. Sung improvisation provides an opportunity to test whether individuals with congenital amusia demonstrate tonal knowledge without outside influence, i.e., in the absence of tonal induction.

The possibility of observing implicit knowledge without awareness in singing comes from an early case study of amusia acquired through brain damage. Peretz^29^ observed that the case GL spontaneously sang a “totally unrelated and unrecognizable tune” (p. 31) when he could not recall the familiar songs asked of him. The individual tones in these improvisations were judged by two experts to be more consistent with a single key (more tonal) than were the tones produced in the remembered songs. Yet, GL was unable to use tonal knowledge in perceptual tasks that involved interpreting melodic closure, discriminating melodies, or aesthetic preference for notes conforming to a scale. These findings suggest that implicit tonal knowledge shapes production in the absence of explicit knowledge. Whether similar results are observed in individuals who exhibit a lifelong deficit in explicit processing of tonal material will be assessed in the present study.

The current research focuses on two core aspects of tonality: the tendency to produce or expect notes that conform to a scale, and the tendency to end melodies on the tonic. Participants who represent the lower end of the spectrum of musical ability and have limited access or awareness of tonal knowledge—nonmusicians and individuals with congenital amusia—were asked to invent melodies based on a series of verbal and musical prompts. We expect nonmusicians raised in a culture with Western tonal music to produce improvisations that conform to the hierarchy of that system. Similarly, individuals with congenital amusia with similar musical exposure should produce songs that are more tonally consistent than expected by chance. However, amusic performance may be less stable than controls. Indeed, amusic individuals have no concept of what a deviant note is and hence cannot monitor and correct their performance via auditory feedback^30^.

## Method

### Participants

Eighteen adults with congenital amusia (*M* = 57.0, *SD* = 20.8 years; 12 female, 6 male), and 15 nonmusician controls (*M* = 58.6, *SD* = 21.3 years; 10 female, 5 male) were recruited from a database of individuals who participated in previous research. Details about socio-cultural background were not collected, but all participants were residents of Quebec, Canada. Participants were not told that the study involved singing unless they asked for more information, and no one with amusia declined to participate except those who had relocated. Status as *amusic* or *control* was established prior to the study using the Montreal Protocol for Identification of Amusia^31^. In the course of those evaluations, participants completed both the Brief Assessment of Music Perception^25^ (BAMP; www.peretzlab.ca/online-test/) and the Montreal Battery for Evaluation of Amusia (MBEA)^32^. Both batteries involve listening to short melodies and noticing musical deviations, or deciding whether two melodies are the same or different by a single note shift of 3–7 semitones that changes the scale (or key), intervals, or contour of the melody. Typical listeners detect these changes readily, and the batteries elicit high performance, but performance is markedly reduced for individuals with amusia. As a measure of individual differences, we calculated a composite score of *melodic pitch perception*^33^ by averaging the percentage of correct responses across the five melodic subtests (MBEA: scale, contour, interval; BAMP: scale, off-key; the two scale tests are identical in the two batteries). As a group, participants with amusia were above chance, corresponding to 50% (*M* = 61.8, *SD* = 8.2, range = 44.8–71.8%, *t*(17) = 6.11, *p* < 0.001, Cohen’s *d* = 1.44, 95% CI [57.7, 65.9]), but importantly, all individuals with amusia were more than 3 *SD* below the control group mean in our sample (*M* = 88.4, *SD* = 5.1, range = 79.0–97.3%, see Supplementary Fig. S2).

Participants also completed an acoustical pitch-change detection task. On each trial they heard five identical tones, of which the fourth tone was the same or changed in pitch by some amount (± 25, 50, 100, 200, or 300 cents)^34^. Whether individuals with amusia can perceive subtle pitch changes is relevant to the acquisition of implicit tonality, because inability to detect small pitch changes, such as the semitone distance between a major and minor interval, could compromise the acquisition of the regularities from Western tonal music. Here we report their performance across all changing relative to non-changing trials. In terms of hits minus false alarms (chance = 0%, perfect = 100%), the group with amusia (*M* = 68.8, *SD* = 20.8, range = 35.4–95.6%) performed more poorly than controls (*M* = 94.5, *SD* = 4.7%, range = 86.0–100%), *t*(31) = 4.68, *p* < 0.001, *d* = 1.63, 95% CI [14.47, 36.86]. These results indicate that pitch-change detection thresholds in amusia are higher than in controls (less sensitive), although it should be emphasized that pitch-change detection in invariant sequences is considerably simpler than detecting pitch violations in varying musical sequences.

All participants had 5 or fewer years of music lessons and there was no group difference between those with amusia (median = 0, *M* = 0.4, *SD* = 1.2, range = 0–5 years) and controls (median = 0, *M* = 0.5, *SD* = 1.1, range = 0–4 years), *p* > 0.7. There were no differences in hearing threshold reduction (dB, 0.5–8 kHz) between groups, *t*(29) = 0.80, *p* > 0.4, 95% CI [-6.1, 14.0] (two participants did not complete audiometry).

A secondary group of 32 naive listeners (*M* = 32.8, *SD* = 17.8, range = 18–79 years; 13 female, 16 male, 3 no response) with typical musical training (*M* = 4.8, *SD* = 5.1, median = 2.5, range = 0–14 years) participated in an online task to evaluate excerpts of the renditions.

All participants provided informed consent for study participation. Participants received payment (improvisors) or an opportunity for a raffled gift card (online evaluators). Supplementary audio recordings were selected from individuals who provided consent for publication of audio recordings in an online open-access publication. Procedures were approved by the Comité d’éthique de la recherche en éducation et en psychologie (CEREP), University of Montreal, and all experiments were performed in accordance with relevant guidelines and regulations.

### Improvisation task

**Figure 1:**
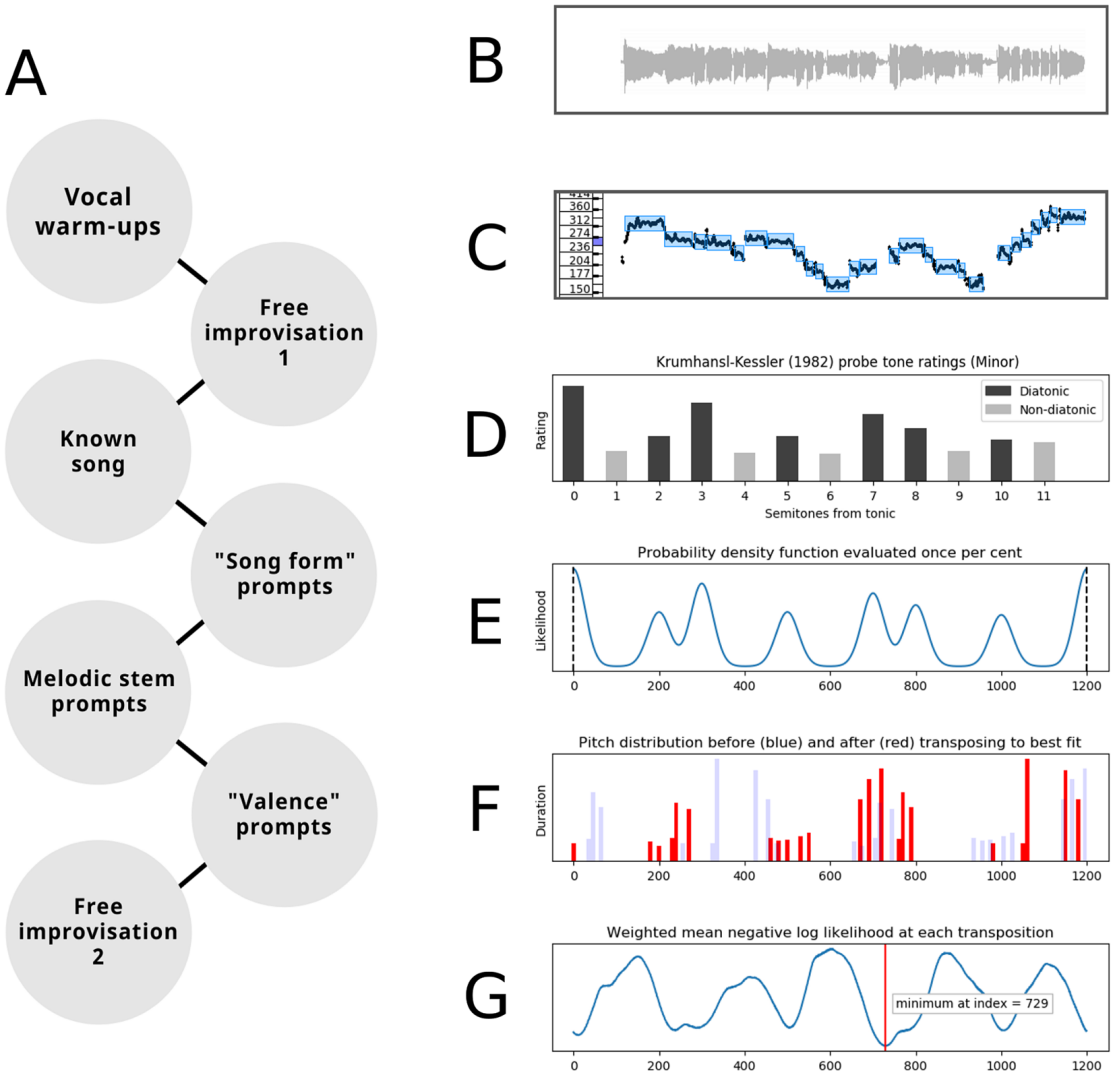
Overview of the task and key-finding algorithm. (**A**) visualizes the general progression of the study. (**B**) visualizes the waveform of an actual improvisation (Audio S1, amusic). (**C**) visualizes the semi-automated annotation of note boundaries (blue boxes) and pitch (black line) in the program Tony. (**D**) visualizes the probe tone ratings (here, minor mode), representing how well each interval fits within the scale, which were used to scale the height and spread of the Gaussians in the normalized probability density function. (**E**) visualizes the normalized probability density function (PDF), which used only the in-scale intervals from (**D**). (**F**) visualizes a histogram of notes from the performance as pitch class in cents (i.e., 0–1200), both in their original value (blue bars) and after being transposed together in steps of one cent, covering the entire octave, until the best alignment with the PDF is found (red bars). By comparing the PDF distribution in (**E**) with the distribution of pitch classes in (**F**), a score of how well the distributions overlap (negative log likelihood) can be calculated for each transposition. (**G**) visualizes the mean negative log likelihood across notes, weighted by note duration, at each transposition. The minimum value indicates the transposition or index with the best fit (vertical red line), which can then be used to locate the tonic to the nearest cent. In short, the algorithm ‘lines up’ the sung notes with the scale as much as possible. The algorithm is visualized dynamically in Supplementary Materials (Movie S1) and available as a python module (see Code Availability).

The task was to sing improvisations in response to different verbal prompts or as extensions of short melodic stems. Figure 1A displays an overview of the task. Improvisations were explained to be “making up a new melody,” which was (1) different from any melody the participant already knew, and (2) different from any previous attempts. Participants were instructed to sing without lyrics, using the syllable ‘*da*’ to distinguish each note (i.e., without gliding between notes). Participants were encouraged to sing for a length of time that felt appropriate to them, but instructed not to pause or restart in the middle of an improvisation. The experimenter was available throughout the session to answer questions and monitor compliance. After each trial, the experimenter asked the participant if they deviated from the protocol. Deviations noticed by the participant (self report, *n* = 17 of 924 trials or 1.8%) or the experimenter (*n* = 22 of 924 trials or 2.4%) were discarded and the trial was repeated.

As described in Fig. 1A, participants performed vocal warm-ups by gliding across their vocal range. The task began with a free improvisation (i.e., no prompt). Next, participants sang a familiar song (*Happy Birthday* or *Gens du Pays*, Supplementary Fig. S3) with lyrics in English or French, and again without lyrics (*da da*). The familiar songs were not included in the improvisation analyses, and were included to evaluate singing ability, as in prior research^35–37^. Participants then improvised four melodies based on song forms common across cultures^38^: a lullaby, a dance song, a love song, and a healing song. Next, participants improvised 16 melodies as extensions of short melodic stems (3 or 4 notes), as well as two melodies based on longer stems (10 notes). For these 18 trials, participants were asked to repeat the stem (shorter stems) or the final notes (longer stems) in their vocal range and continue it. A short example, recorded by the vocalist who recorded the stimuli, preceded the trials. For trials with longer stems, participants were asked to improvise an extension of the last few notes, although some participants attempted to repeat more of the melody.

The 18 melodic stems (Supplementary Fig. S4) were composed in pairs. One version was composed to elicit a greater sense of tonality, the other a lesser sense, while keeping the same contour, although the short stems were unlikely to impose a strong sense or absence of tonality. Due to an error, the eighth short tonal stem was presented twice in place of the less tonal version. In total, there were 10 trials with more tonal stems, and 8 trials with less tonal stems. The stems were recorded by an amateur female singer using the syllable ‘*da’* and pitch-corrected in Melodyne (Celemony, Inc.). The stems were normalized (RMS) and saved as high-quality digital audio files (48 kHz, 16-bit). They included notes A3 to A4 (i.e., 220–440 Hz). Most intervals were small (< 4 semitones), and all were 7 semitones or less, requiring no particular effort.

Following the 18 musical prompt trials, participants were asked to improvise two happy and two sad songs, in alternating order. Finally, the session concluded with a second free improvisation. In total there were 30 recordings per participant: 28 improvisations and 2 familiar songs. Participants performed all trials in the same order, and the primary analyses considered all improvisations as a set (e.g., average scores), reducing the potential impact of order effects. The recording session lasted approximately 30–40 min.

Participants were tested individually in a sound-attenuating booth, while seated in front of a computer running Audition software (Adobe, Inc.), which controlled presentation of melodic prompts via headphones (DT-770 Pro, Beyerdynamic, Inc.) and recorded improvisations via microphone (Neumann TLM-103) as high-quality digital sound files. An experimenter controlled the program from outside the booth and communicated through the participant’s headphones. Volume was adjusted to a comfortable level for each participant.

### Analyses

#### Outline

We first considered whether the characteristics of improvisations differed by group in terms of length, number of notes, pitch range, or number of pitch classes, which could indicate qualitatively different approaches to the task. The primary, hypothesis-driven analyses were assessments of how well each rendition adhered to the scales of Western tonal music (i.e., major and minor scales), whether the groups differed from each other (independent-samples t-tests) and from chance (one-sample t-tests), and whether individuals differed from chance (one-sample t-tests, binomial tests). As detailed below, tonality was assessed as the proportion of notes “in-tune” or “in-scale”, as well as the tendency to end a rendition on the tonic of the scale. We considered as well whether the more or less tonal melodic stems would influence the tonality of the improvised continuation.

The results also include exploratory analyses using ratings from a group of naive listeners (see Participants). In the first analysis, they evaluated enjoyment of the task via smiling assessments. These were used as an implicit measure of enjoyment, because smiling tends to indicate positive affect, and because naive listeners can successfully differentiate auditory tokens with both smiling and unsmiling acoustic features^39^. Enjoyment was assessed in this indirect and implicit manner because it was not assessed directly during the original session and it is relevant to the wider use of this paradigm (e.g., children). In the second exploratory analysis, the same naive listeners attempted to identify the intended song form of improvisations which were directed to be a lullaby, dance song, healing song, or love song. These forms are widely produced and widely recognizable across cultures^38^, likely due to a combination of cues not limited to the tonal encoding of pitch.

#### Pre-processing

Continuous pitch (Hz) and note boundaries were generated automatically for each recording using the program Tony^40^, and checked manually for transcription errors (Fig. 1B–C). Impossibly short notes (< 50 ms) were excluded automatically. In some instances (33 of 924 improvisations, or 3.6% of total trials), the recording was truncated because participants began singing before the record button was pressed, or they sang indefinitely, forcing the experimenter to end the trial. Trials ended early by the experimenter were excluded from analyses of the final note.

A central pitch value for each note was calculated as the median of the continuous frequency values within the middle 50% of the note. The pitch value was transformed from frequency (logarithmic) to semitone (linear) using A440 as reference (i.e., standard MIDI values, where 60 = C4). Each value was calculated to the nearest cent (i.e., two decimals, 100 cents per semitone). In rare instances, the pitch-tracking algorithm in Tony (pYIN) was indeterminate for more than half of a note due to recording or voicing quality, and that note alone was excluded (0.12% of total notes). From the remaining notes, intervals in pitch were calculated for each pair of adjacent notes to the nearest cent.

#### Determining the nearest key

In contrast to tones produced by instruments with stable, discrete pitches (e.g., piano), sung tones are rarely aligned perfectly with equal temperament tuning (i.e., 440 Hz reference pitch), even among professional vocalists. We developed an algorithm to determine the nearest tonic/key using continuous pitch values (i.e., to nearest cent). Pitch values were compared to the in-scale intervals of the major and minor ratings of probe tone tasks^8,16^ using a probability density distribution (PDF; see Fig. 1D–E) to reveal tonal relations among sung pitches. The PDF was built by summing a series of Gaussian curves with means centred on intervals in the scale. The spread of all Gaussians (i.e., *SD*) was determined by calculating the pitch stability (i.e., *SD*) within notes, averaged across all notes sung in the study (40,032 notes, *M* = 26.1 cents). Gaussians were evaluated at steps of 1 cent across the octave, resulting in 1200 values which were then normalized to sum to 1. Together, they represent a probability for pitches in-scale. MIDI values were converted to pitch class using modulo arithmetic (e.g., mod 12 of MIDI note 65.45 becomes 5.45), then converted to cents (e.g., 5.45 * 100 = 545), and the negative log likelihood of the distribution at that index (e.g., index 545 of 1200) served as a measure of fit for that tone. In turn, the *mean* of negative log likelihoods for all tones in an improvisation, weighted by relative duration, measured the fit of the improvisation as a whole. The entire melody was then transposed in steps of 1 cent (1200 times) to find the best fit across the octave, and the index at that transposition was used to determine the tonic (see Fig. 1F–G). For every improvisation, this procedure was completed for both the major and minor scale profile (best fit of 2 * 1200 = 2400 comparisons total) to determine the tonic and mode to the nearest cent.

#### Proportion of tonal notes

The proportion of notes in the rendition that were tonal was calculated using the determined key. For example, in the key of C-major, notes in the list [C, D, E, F, G, A, B] would be marked as in-scale and notes in the list [C#, D#, F#, G#, A#] would be marked as out-of-scale. Individual notes were marked as tonal if they were less than 50 cents from an interval that was in-scale.

Because chance performance depends on the number of notes (see Supplementary Fig. S5), a null distribution was calculated for every improvisation: (1) the pitch of each sung tone was reassigned at random (i.e., preserving durations), (2) the key-finding algorithm located the nearest key and mode given the random pitch values, (3) the proportion of randomized notes that were in-key relative to that key and mode was logged, and (4) the process was repeated for a total of 1000 iterations. The M and SD from the null distribution were used to calculate a z-score of the proportion of tonal notes in the actual improvisation (z = [actual–M]/SD), such that a score of 0 approximates chance regardless of number of notes. Musicians' evaluations of the tonal coherence of a sample of improvisations correlated with the results of the algorithm (Supplementary Fig. S6).

The null distribution from which pitches were sampled was uniform across the octave. Changing the shape of the null distribution to match the range of each performance from minimum to maximum pitch (‘uniform-range’; z-score mean difference = − 0.16) or to match the range *and* central tendencies (*M*, *SD*) of the performance (‘Gaussian-range’; z-score mean difference = − 0.02) had a minimal effect on z-scores in the context of the overall distribution (z-score *SD* = 1.78). Moreover, scores generated by those alternative distributions correlated highly with the analysis reported here and with one another (*r*s > 0.99), and the outcomes of statistical tests reported in results did not differ.

#### Evaluation of smiling

Auditory excerpts were the first 5 s of each free improvisation (i.e., no prompt) and each familiar song without lyrics (i.e., *Happy Birthday* or *Gens du Pays*). Additionally, participants rated excerpts of two amateur singers who were directed to sing *Happy Birthday* with a smiling or neutral face (baseline). Each listener rated the same 15 items from each baseline category, and 51 items sampled from the target excerpts, on a scale from 0 (*"no smile"*) to 10 (*"a lot of smile"*). Both the singers of baseline stimuli and the listeners were naive to the purpose of the study.

#### Evaluation of song form

The same evaluators participated in a second task to identify the intended song form for trials with those verbal prompts (see Improvisation Task). Due to time constraints and differences in rendition length, auditory excerpts were restricted to the first 5 s of each improvisation elicited from a song form prompt. Each listener evaluated 20 improvisers sampled at random. Hence the number of ratings varied from improviser to improviser, and from excerpt to excerpt. Thus, some improvisers were evaluated more than others (median = 10, range = 4–15 evaluations). Responses were forced-choice (*lullaby, dance song, love song, healing song*).

Examples of improvisations which received high and low scores in the tonality analysis are available in Supplementary Information (Audio S1–S4). All statistical tests are two-sided unless otherwise noted. Non-parametric tests converged on the same results as the parametric tests reported here.

## Results

**Figure 2:**
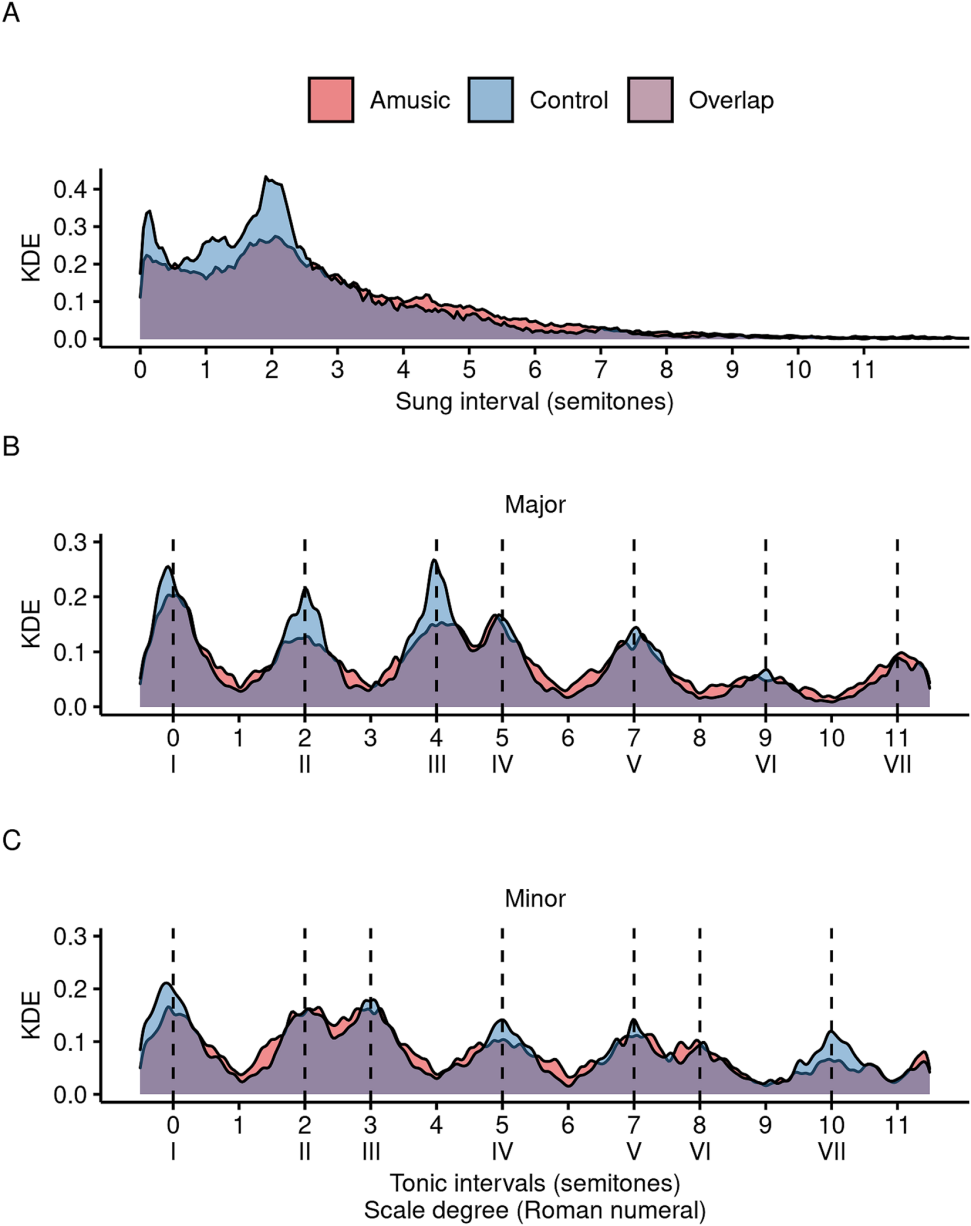
(**A**) shows the distribution of sung intervals (comprising both ascending and descending intervals) in improvisations by group. Intervals beyond 12 semitones were infrequent and are omitted for clarity. (**B**) and (**C**) show the density plot of all notes sung during improvisations relative to the tonic. Vertical dashed lines represent intervals in-scale by mode. Supplementary Fig. S7 displays panels (**B**) and (**C**) alongside a density plot derived from the null distribution used in the z-score analysis. KDE = Kernel density estimate, an estimate of the probability density function; kernel bandwidth = 1/10.

Characteristics of improvisations are summarized in Table 1. Both groups produced on average more than 30 notes in each improvisation. There were no differences between groups in median number of notes, *t*(31) = 0.12, *p* > 0.9, *d* = 0.04, 95% CI [− 12.50, 14.04], nor median duration in seconds, *t*(31) = 0.90, *p* > 0.3, *d* = 0.31, 95% CI [− 4.5, 11.6], suggesting that both groups approached the task with a similar degree of enthusiasm. Both groups also favored small intervals, with 56.5% being smaller than |250| cents (see also Fig. 2A), as is typical in most music^41^, and controls used small intervals more than did participants with amusia, *t*(31) = 2.65, *p* = 0.013, *d* = 0.93, 95% CI [0.028, 0.215]. There was no difference between groups in the number of unique pitch values sung per improvisation, *t*(31) = 0.92, *p* > 0.3, *d* = 0.32, 95% CI [− 0.51, 1.34]. The proportion of improvisations determined to be in the major mode differed by group, *t*(31) = 3.90, *p* < 0.001, *d* = 1.36, 95% CI [0.085, 0.271]. Analysis of the algorithm revealed that the group with amusia did not differ from chance (proportion major = 0.459), *t*(17) = 1.14, *p* > 0.2, *d* = 0.27, 95% CI [0.438, 0.531]. Rather, the control group sang more in major keys than expected by chance, *t*(14) = 4.77, *p* < 0.001, *d* = 1.23, 95% CI [0.570, 0.753], in line with historically greater prevalence of major mode in popular music^42^.

**Table 1:** Improvisation characteristics by group.

Characteristics of improvisations			
Median or proportion ± IQR (Min–Max)			
	Amusic	Control	Group difference
N	18	15	
Median number of notes	34.2 ± 14.0 (15.0–77.0)	31.5 ± 18.2 (15.5–97.0)	–
Median duration (s)	22.9 ± 6.5 (12.0–62.1)	19.7 ± 8.1 (11.3–42.3)	–
Median pitch range (semitones)	11.9 ± 2.8 (8.3–20.7)	11.7 ± 3.5 (7.7–17.2)	–
Median number of pitch classes	10.0 ± 2.0 (8.0–12.0)	10.0 ± 1.0 (7.0–12.0)	–
Median prop. small intervals (<|250| cents)	0.49 ± 0.23 (0.33–0.76)	0.68 ± 0.19 (0.44–0.81)	*
Proportion of major mode	0.46 ± 0.10 (0.36–0.68)	0.68 ± 0.16 (0.39–0.96)	*
Proportion of tonal notes	0.72 ± 0.09 (0.69–0.85)	0.83 ± 0.08 (0.74–0.96)	*

Scores were summarized by individual (28 trials) prior to group-level summary statistics displayed here. Dash indicates not significant, asterisk indicates *p* < 0.05.

The distribution of pitches relative to the tonic (tonic interval) is a signature of the tonal hierarchy. The global distribution of tonic intervals for individual notes (*n* = 38,131), is visualized separately by group and whether the algorithm labeled the rendition as ‘major’ (Fig. 2B) or ‘minor’ (Fig. 2C) but pooled across participants and trials (*n* = 9979 amusic/major; *n* = 10,951 amusic/minor; *n* = 11,256 control/major; *n* = 5945 control/minor). The difference in density distributions by mode (upper versus lower panel) confirms that the algorithm returned results that approximated the a priori distribution of in-scale intervals used in the algorithm (i.e., distance between peaks), and to a lesser degree, the probe tone ratings used to build those distributions (i.e., relative peak heights; see Supplementary Fig. S8 for direct comparison in controls). The vast majority of both major and minor distributions overlapped across groups (purple shading). For several in-scale intervals, controls show clearer ‘peaks’ (e.g., blue shading at major mode intervals of 0, 2, or 4 semitones) and ‘valleys’, collectively indicating greater adherence to the scale in controls.

Additional analyses included in Supplementary Information rule out the possibility that participants with amusia were more reliant on simple pattern repetition (Supplementary Fig. S9) and replicate previous findings of reduced singing accuracy in amusia (Supplementary Fig. S10).

### Tonality

**Figure 3:**
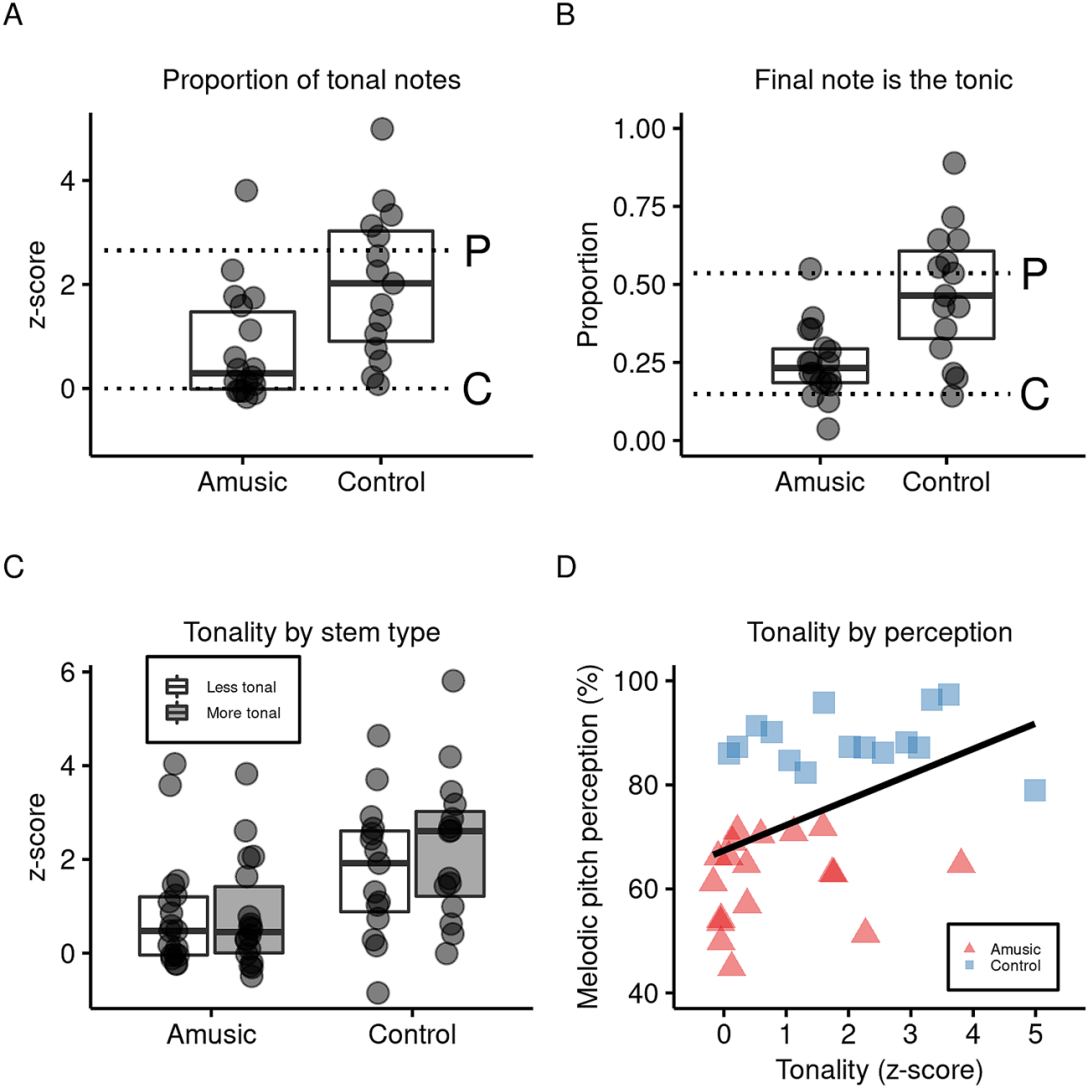
Tonality of improvised songs. (**A**) shows the z-transformed proportion of tonal notes. Individual data points were calculated as the average of 28 improvisations, which were z-transformed against 1000 randomly generated melodies. The upper dashed line represents the score of a professional baritone singer with 11 years of formal training and 29 years of total singing experience. His data provide a benchmark of proficient singing since the measures are standardized with a clear baseline but no clear ceiling. The lower dashed line represents chance, i.e., z-score of 0. Both groups were above chance and the control group had higher scores than the group with amusia. (**B**) shows the proportion of trials in which the participant ended their improvisation within 50 cents of the tonic determined by the key-finding algorithm. Each value was calculated as an average from 28 improvisations, except in a handful of cases with truncated recordings. The upper dashed line represents the score of a professional singer. The lower dashed line represents chance (0.149), as estimated from over one million random permutations generated over the course of the analyses. Both groups were above chance and the control group had higher scores than the group with amusia. (**C**) shows mean tonality of improvisation as measured by proportion of tonal notes (z-score) according to group and type of stem. An interaction between group and stem was driven by a significant difference between more tonal over less tonal stems in the control group only. All subgroupings of the data (i.e., individual boxplots) were significantly above chance (i.e., z-score of 0). Boxplots display median (line) and 25th and 75th percentiles (box edges). (**D**) shows individual melodic perception scores (see Participants) plotted against the tonality of improvisations. The solid trend line visualizes a significant positive correlation across all participants.

In order to measure the tonality of each improvisation, a z-score was computed for the proportion of tonal notes relative to chance in each improvisation, as detailed above. The z-scores obtained from all 28 improvisations were averaged into a single score for each participant. As visible in the individual data points in Fig. 3A, each group showed a wide range of performance, including individuals near chance in the control group, and individuals in the group with amusia near or above the control group median. Despite the large variability, an independent-samples t-test revealed a significant difference between groups, *t*(31) = 2.90, *p* = 0.007, *d* = 1.02, 95% CI [0.37, 2.14]. Both the group with amusia, *M* = 0.77, *SD* = 1.08, *t*(17) = 3.01, *p* = 0.008, *d* = 0.71, 95% CI [0.23, 1.30], and the control group, *M* = 2.02, *SD* = 1.41, *t*(14) = 5.56, *p* < 0.001, *d* = 1.43, 95% CI [1.24, 2.81], had significantly better performance than chance. On an individual level, i.e., separate one-sample t-tests per participant (*df* = 27), performance was significantly above chance (z-score of 0, *p* < 0.05) for 7 of 18 individuals with amusia and 13 of 15 in the control group. Thirteen of 18 individuals with amusia, and all controls, had a positive average z-score in absolute terms.

In addition, the proportion of all 28 improvisations that ended on the tonic was calculated for each participant, excluding instances of truncated recordings (Fig. 3B). An independent-samples t-test revealed a significant difference between groups for proportion of final notes within 50 cents of the tonic, *t*(31) = 3.90, *p* < 0.001, *d* = 1.36, 95% CI [0.107, 0.341]. We used the null distributions (*n* = 1,020,000) generated for the z-scores to examine the proportion of times that the final note matched the tonic by chance. Melodies composed of random notes ended on a note within 50 cents of the tonic identified by the PDF algorithm 0.149 proportion of the time. Both the group with amusia, *M* = 0.25, *SD* = 0.12, *t*(17) = 3.64, *p* = 0.002, *d* = 0.86, 95% CI [0.191, 0.306], and controls, *M* = 0.47, *SD* = 0.21, *t*(14) = 6.01, *p* < 0.001, *d* = 1.55, 95% CI [0.357, 0.587], had significantly higher proportions than chance, despite wide inter-participant variability. On an individual level, i.e., separate one-tailed binomial tests per participant (*df* = 27), performance was significantly above chance (proportion > 0.149, *p* < 0.05) for 6 of 18 individuals with amusia and 12 of 15 in the control group. Fifteen of 18 individuals with amusia, and 12 of 15 controls, had an average proportion above 0.149 in absolute terms.

Next, the tonality of improvisations in response to musical stems was examined with a mixed-model ANOVA with group (amusic, control) as a between-participant factor and stem (more tonal stem, less tonal stem) as a within-participant factor (Fig. 3C). There was a significant main effect of group, *F*(1, 31) = 6.95, *p* = 0.013, *η*_*p*_^*2*^ = 0.18, replicating the previous analyses showing more tonal performance in the control group. There was also a main effect for stem, *F*(1, 31) = 4.54, *p* = 0.041, *η*_*p*_^*2*^ = 0.13; however these main effects were qualified by an interaction, *F*(1, 31) = 5.82, *p* = 0.022, *η*_*p*_^*2*^ = 0.16. Follow-up pairwise comparisons (Bonferroni-Holm) showed that the advantage of tonality for only one comparison—more tonal stems versus less tonal stems—reached a statistically significant difference in controls, *t*(14) = 4.05, *p* = 0.003, *d* = 0.31, 95% CI [0.220, 0.736], but not in participants with amusia, *t*(17) = 0.18, *p* > 0.8, *d* = 0.02, 95% CI [− 0.315, 0.375]. Whereas controls tended to sing more tonal improvisations when starting from tonal versus atonal stems, there was no effect of stem tonality in the group with amusia. Nevertheless, for both groups, more tonal and less tonal stems were above chance in tonality (Bonferroni-Holm), *p*s < 0.05. Additionally, in both groups the improvisations sung as extension of the longer tonal stem (amusic: *M* = 0.52, *SD* = 1.66; control: *M* = 1.96, *SD* = 1.98) was not more tonal than the average score for short tonal stem trials (amusic: *M* = 0.86, *SD* = 1.17; control: *M* = 2.30, SD = 1.51).

The ability to detect pitch changes of 25–300 cents in an acoustic context (see Participants) did not correlate with mean tonality of improvisations across all participants, *r*(31) = 0.06, *p* > 0.7, 95% CI [− 0.29, 0.39]. In contrast, the relationship between *musical* pitch perception (i.e., mean of tonal subtests of BAMP and MBEA) and mean tonality of improvisations was positive across all participants, *r*(31) = 0.44, *p* = 0.009, 95% CI [0.12, 0.68] (Fig. 3D). The latter relationship was not, however, significant for either group when examined separately, *p*s > 0.5.

### Evaluation of smiling

**Figure 4:**
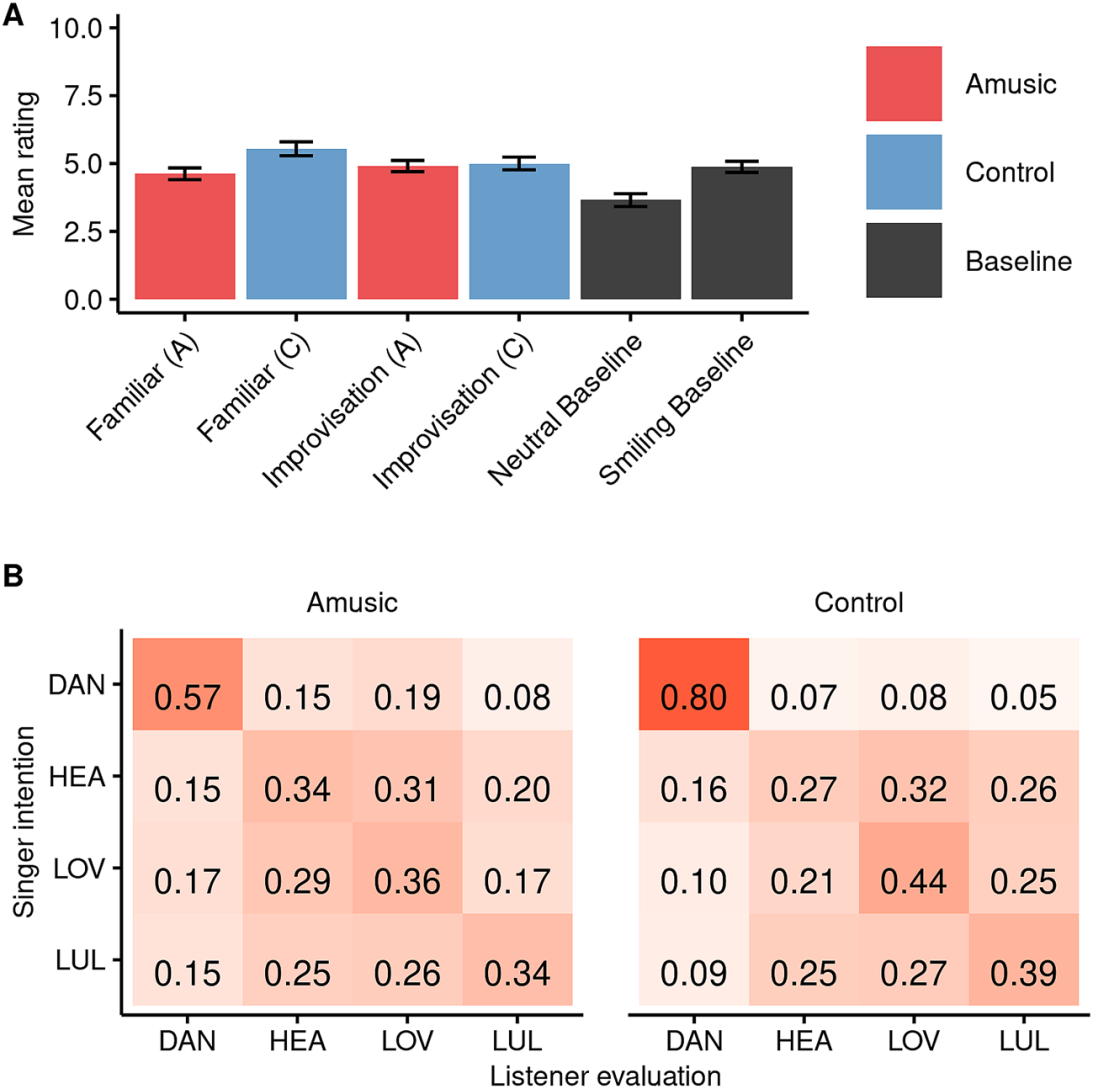
Evaluations of brief singing excerpts (first 5 s) by naive listeners. (**A**) shows average ratings on a scale from 0 (*"no smile"*) to 10 (*"a lot of smile"*) for excerpts of the familiar song (i.e., *Happy Birthday* or *Gens du Pays)* or free improvisations, separately by singer group (amusic, control). Two amateur singers who sang *Happy Birthday* with a smiling or neutral face served as baseline. All categories scored significantly higher than the neutral baseline recordings, and were not lower than the neutral smiling recordings, consistent with implicit enjoyment of the task. (**B**) shows a confusion matrix for improvisation excerpts sung in response to a prompt to sing a dance song (DAN), a healing song (HEA), a love song (LOV), or a lullaby (LUL), separately by singer group (amusic, control). Scores along the descending diagonal are correct evaluations, and were more accurate than chance (proportion = 0.25) for all forms sung by individuals with amusia, and three of the four forms (dance song, love song, lullaby) sung by control participants.

For each evaluator, ratings were averaged by category (familiar song, improvisation) and singer group (amusic, control) (see Fig. 4A). Planned contrasts (Bonferroni-Holm) compared the ratings to baseline. All categories were rated as significantly more "smiling" than the neutral baseline, *p*s < 0.001. No categories were rated as less "smiling" than the smiling baseline, *p*s > 0.5, and only one category (familiar-control) elicited significantly higher ratings than the smiling baseline, *t*(31) = 3.13, *p* = 0.003, *d* = 0.50. Moreover, there was no difference in the evaluation of smiling between amusic and control improvisations, *p* > 0.4. By these implicit measures, both amusic and control singers found the task enjoyable.

### Evaluation of song form

For each evaluator, the proportion of correct evaluations was calculated as 8 scores grouped by category (dance song, love song, healing song, lullaby) and singer group (amusic, control), with a range of 4–15 ratings averaged per score. Figure 4B displays a confusion matrix pooled across all ratings. Each of the 8 categories was compared to the chance proportion correct (0.25) in a series of Bonferroni-Holm corrected one-sample t-tests (*df* = 31). All tests were significantly above chance, *p*s < 0.002 (range of* M* = 0.34–0.80), except for the category of healing songs performed by control participants (*M* = 0.27). In line with previous research^38^, dance songs were the least likely to be confused with other forms. In a series of four paired t-tests (Bonferroni-Holm), evaluations were more accurate for control than for amusic dance songs, *t*(31) = 7.19, *p* < 0.001, *d* = 1.20, and more accurate for amusic than for control healing songs, *t*(31) = 2.65, *p* = 0.038, *d* = 0.53, but did not differ by singing group for love songs or lullabies, *p*s > 0.1. Overall, the results show successful evaluation of song forms from brief excerpts (5 s) of improvisations. They also show that singers with amusia can accurately convey internal models of song forms.

## Discussion

The current study shows that improvisation can serve as a novel tool to study tonality, which is a core component of musicality in tonal music. Even some of the poorest singers invent songs with pitch variations conforming to the tonal hierarchy of Western music and following recognizable forms. Moreover, the task is surprisingly straightforward and approachable, even enjoyable. The main benefit of using improvisations over perceptual or other production tasks is that measures of implicit knowledge of music can be collected directly and without guiding the behavior of interest.

Tonal organization of pitch is traditionally assessed by induction^15^. We provide the first evidence that two core aspects of tonality—tonal hierarchy of pitches and resolution on the tonic—occur in the absence of tonal induction. The observation of tonality in freeform productions suggests that abstract rules guide adult musicality. These rules were assessed over the rendition as a whole, with median length over 20 s and 30 notes, far exceeding the limits of short-term memory. Such stability showcases the robustness of tonal schemas. Moreover, signatures of tonality were observed in most typical nonmusicians and in a subset of individuals with amusia, which supports the view that awareness is not required for acquiring and sustaining tonal organization over time. In short, even some of the least musical among us spontaneously produce the learned tonal regularities of music.

However, expressions of tonality varied. Some individuals in both the amusic and control groups were above both group medians (more tonal), and some were below both group medians (less tonal), with considerable overlap between groups. Nevertheless, those with amusia produced less tonal improvisations than did nonmusicians overall. Groupwise differences in tonality could not be explained by limitations in pitch range (lowest to highest pitch) or enthusiasm (duration, number of notes, ‘auditory smiles’), and their distribution of intervals was similar to what has been reported in various corpora of Western music^41^. A major question for future research is whether the propensity for tonality in improvisations reflects lifetime listening experience or predispositions to produce specific distributions of pitches. The current data did not include detailed listening histories or diaries, but we note that the screening test for amusia included the question, *‘do you intentionally listen to music?’* (*1—never*, *5—very often*) which significantly correlated with improvisation tonality (z-score), *r*(31) = 0.38, *p* = 0.031 (Spearman). This suggests a significant contribution of listening experience. In any case, the findings of groupwise differences and overlap across groups suggest that congenital amusia leads to a difference in tonal knowledge as a matter of degree rather than category.

Uncovering the origins of this difference, especially in populations who cannot readily correct errors, is a key to harnessing optimal learning conditions. Congenital amusia is a model condition for delineating those conditions because musical knowledge is built in isolation from tutoring, awareness, and feedback. Peretz^30^ proposed that deficits in amusia, like other congenital disorders of awareness (prosopagnosia, dyslexia), may indicate sparse knowledge that is functional only at an implicit level. This suggests that awareness is necessary to shape musical knowledge acquired by mere exposure to enjoy a normally functioning system.

The role of awareness is hard to distinguish from feedback in auditory-vocal learning. The auditory feedback system serves a major role in monitoring and adjusting errors. If altered, a poor calibration occurs between the implicitly learned (bottom-up) knowledge in the auditory cortex and the (top-down) control of that knowledge from frontal brain areas, as modeled in Peretz^30^. As a result, a poor assessment of whether a note fits the preceding context, as in the melodic subtests of the MBEA^32^, occurs, as observed here. Across all participants, there was a positive correlation between performance on those subtests and tonality of improvisations. Thus, the results are consistent with a poor feedback loop. As a matter of fact, it is relatively easy to alter the detection of an out-of-scale note by manipulated feedback in typical listeners^43^. In that study, nonmusicians were asked to evaluate whether a melody contained a “wrong” (out-of-scale) note and received audiovisual feedback about whether they were correct. Unbeknownst to participants, the researchers manipulated whether the feedback was random or accurate. In the accurate condition, participants demonstrated typical behavioral and neurophysiological responses to out-of-scale notes, but in the random feedback condition, both types of measures showed responses more typical of amusia. Whether task feedback can, in turn, be used to train individuals to overcome their deficit is a key question for future research.

Fortunately, improvisation can bypass individual limitations in learning and is arguably the most suitable method to capture individual variation in musical knowledge representations. The present study of tonality is a proof of concept that improvisations can serve as a rich source of data for exploring the internal architecture of musicality. It is also an accessible method. Microphones built into computers or phones produce adequate recordings for analysis and are readily available, and task instructions are intuitive and straightforward. Improvisation could reveal many other aspects of musicality. One important building block of tonal music concerns temporal regularities and structures. For example, to what degree do improvisations show the presence of a steady beat^44^, or simple integer subdivisions of the beat^45^? Furthermore, improvised music could be used to train or compare computational models of expectancy that are used widely in psychology and neuroscience of music^13,46^. We envision limitations and pitfalls to the method as well. Even with modern software, singing analysis can be tedious and error-prone, and the demarcation of note boundaries becomes difficult in singing with lyrics, expressive singing, or in singing with gliding pitches. Nevertheless, we envision that the method of improvisation, which places few constraints on the participant and uses the ‘universal’ instrument—the voice—could facilitate cross-cultural and cross-species research on regularities in music, such as comparing songs across developmental periods (*when do children acquire the rules of music?*), across cultures (*what rules are universal?*), or even across species (*what rules are unique to humans or shared with other vocal learners?*). The present data form the basis for a novel corpus that will be a springboard for such analyses.

Finally, we were pleased to discover that participants with amusia improvised enthusiastically and even demonstrated ‘auditory smiles’^39^. One participant with amusia later communicated that she enrolled in singing lessons because she enjoyed her experience in the study. Other typically shy populations, such as young children, might find improvisation more approachable than other production tasks because it is a natural activity. Indeed, given its prevalence as an everyday behavior, future research might study the origins of reward and pleasure from spontaneous music making, as well as its physiological effects^47^ and potential as a therapeutic tool^48^.

## Supplementary Information

Supplementary Audio 1.

Supplementary Audio 2.

Supplementary Audio 3.

Supplementary Audio 4.

Supplementary Video 1.

Supplementary Information 1.

## Data Availability

The datasets generated during and/or analysed during the current study are available from the corresponding author on reasonable request.

## Code availability

The algorithm developed to determine key and measures of tonality is available as a python module on the first author’s GitHub account: https://github.com/m-w-w/pdf_tonality.
