# Supplementary material for: Improvisation is a novel tool to study musicality
Source: Sci Rep. 2022 Jul 22;12:12595. doi: 10.1038/s41598-022-15312-5 (PMC9307610; doi:10.1038/s41598-022-15312-5)
Supplement: Supplementary file 6 — Supplementary Information 1. [file 41598_2022_15312_MOESM6_ESM.docx]

**Fig. S1. Prevalence of spontaneous improvisation in adults.** We
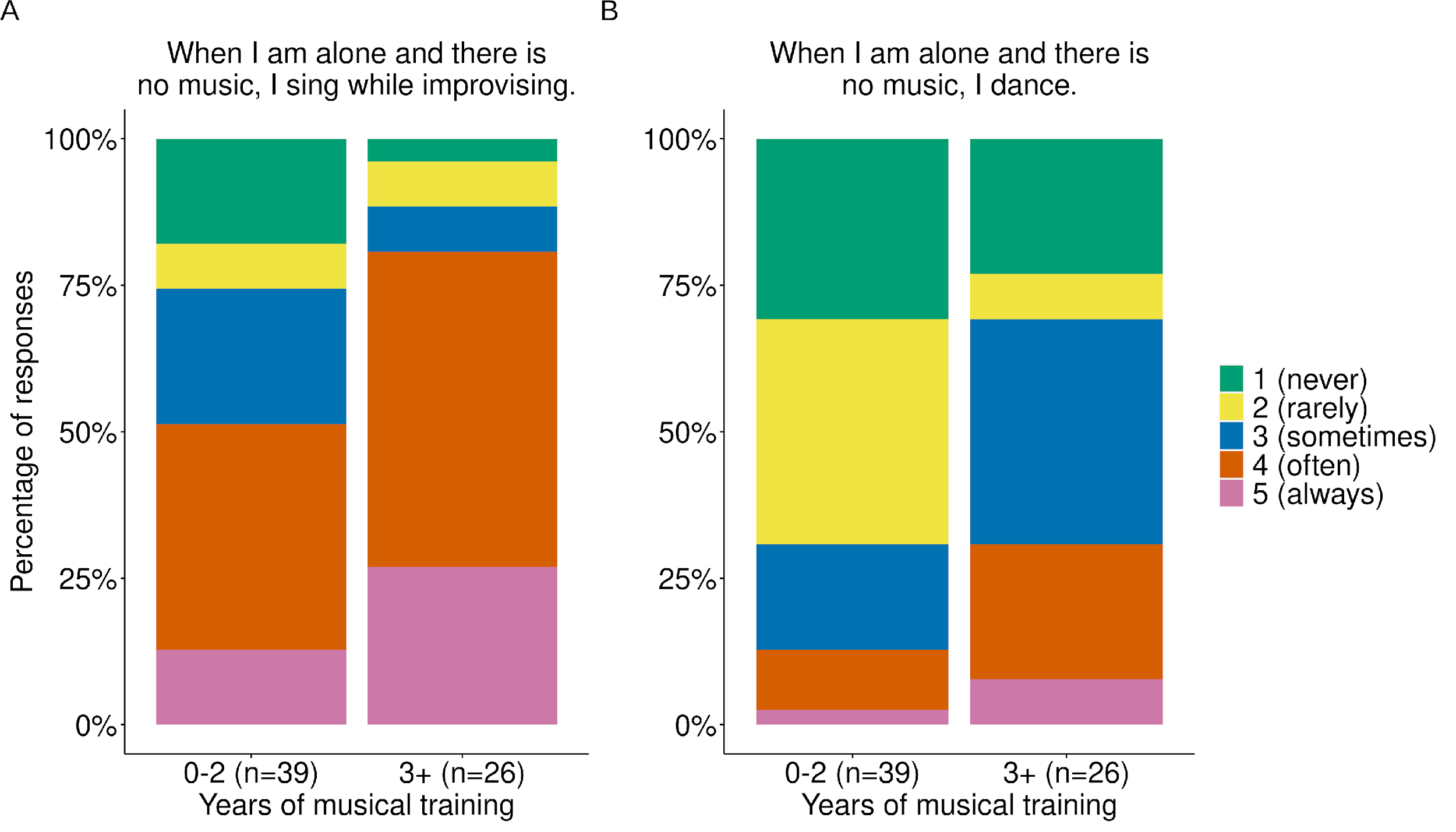
 surveyed 65 undergraduates who were not majoring in a music-related field and had never taken singing lessons. Among the sample, 39 individuals could be considered nonmusicians (0–2 years of lessons) and 26 could be considered more musical or musicians (3+ years of lessons). Respondents were excluded if they majored in a musical field or had taken singing lessons. The prompts asked how often they (1) sang while improvising or (2) danced, in the absence of other people and music, on a scale from 1 (never) to 5 (always). For singing, 74.4% of nonmusician respondents answered “sometimes” (n = 9), “often” (n = 15), or “always” (n = 5), and only 25.6% responded “rarely” (n = 3) or “never” (n = 7). In contrast, the majority of those same nonmusicians (69.2%) reported that they “never” (n = 12) or “rarely” (n = 15) danced in the same conditions. Those with more musical training were more likely to report both behaviors, as might be expected.


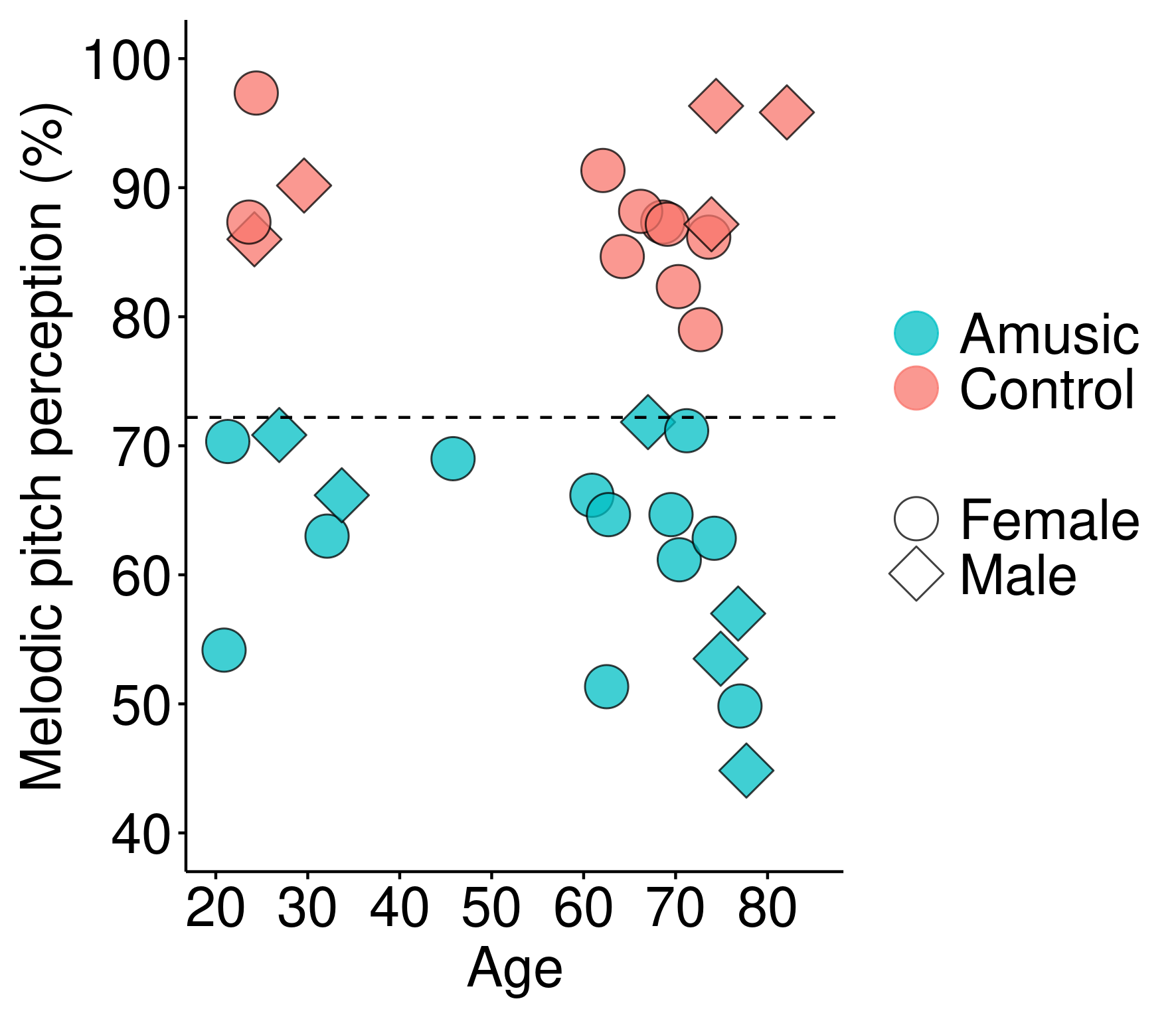


**Fig. S2. Melody perception.** Individual mean scores obtained on five tests requiring detection of a pitch change in melodies from the Brief Assessment of Music Perception (BAMP) and Montreal Battery for Evaluation of Amusia (MBEA) are plotted according to age and gender of participant. Chance performance is 50% correct. Both tests are designed to elicit high scores in the general population, and performance below 72.2% (dashed line) is rare and indicative of amusia^1^.


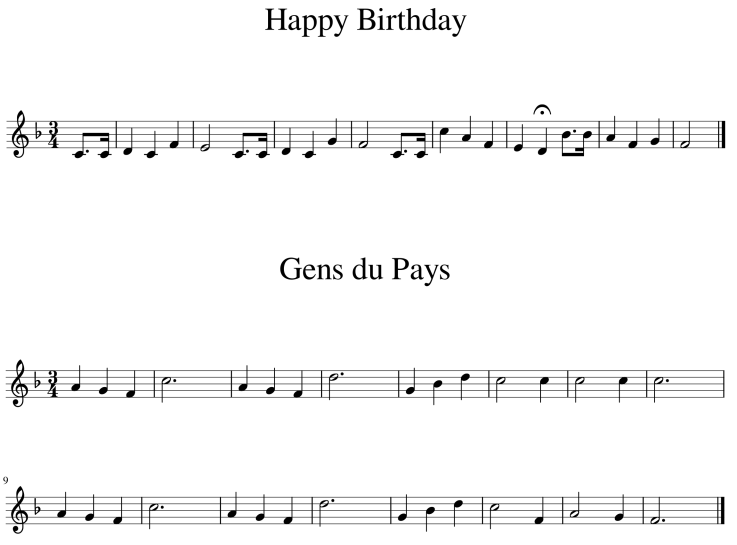


**Fig. S3. Musical notation for the highly familiar songs Happy Birthday and Gens du Pays.** Participants sang one of these melodies twice, with and without lyrics (‘da da’), in addition to improvisations.


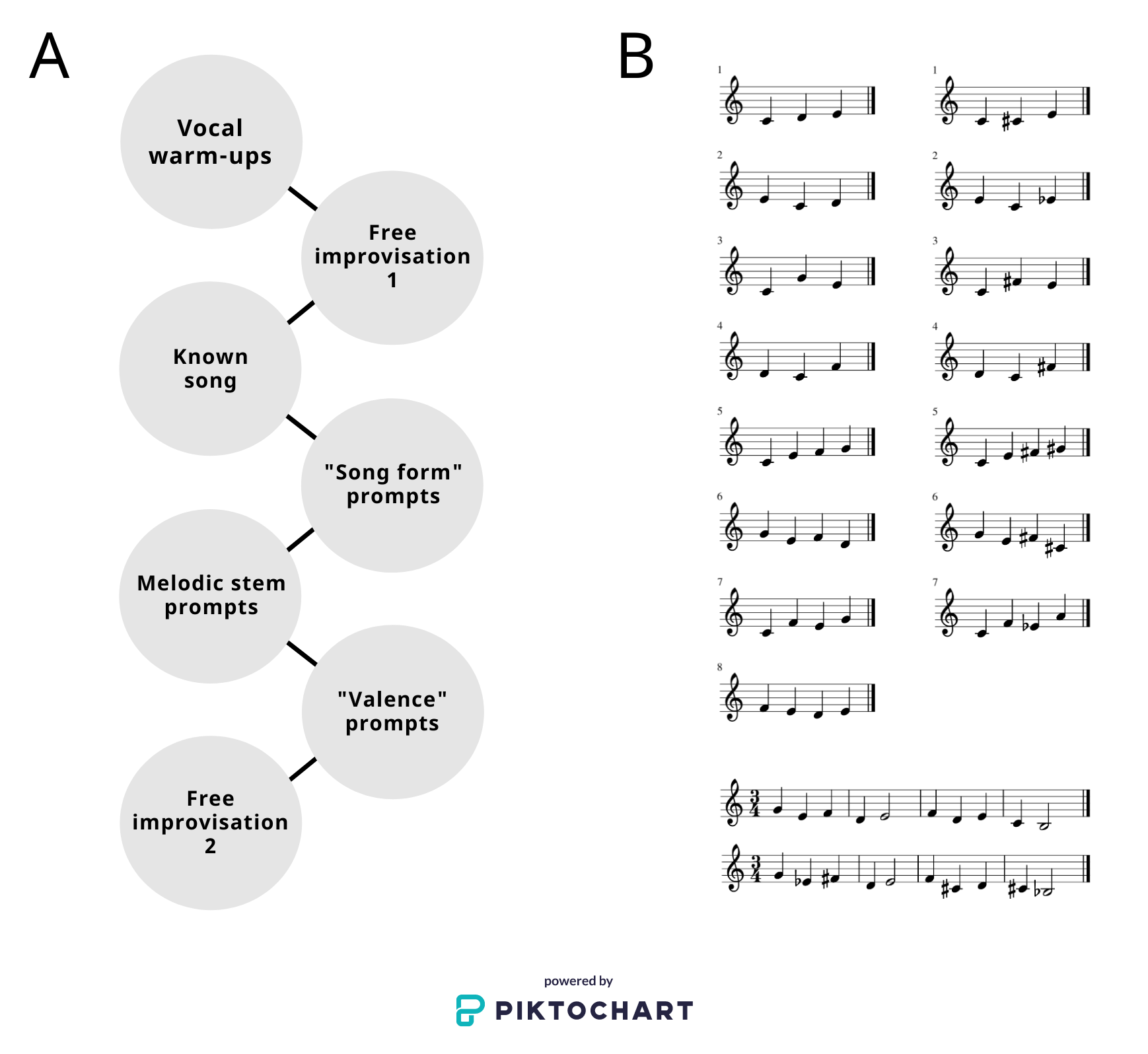


**Fig. S4. Musical notation for the melodic stems used in the study.** Eight short melodic stems were more tonal (left column) or less tonal (right column) versions of the same three- or four-note melodic contour, with the exception of the eighth more-tonal stem, which was repeated due to an error. There were also two long melodic stems, one more tonal (upper) and one less tonal (lower) version of the same melodic contour.

*
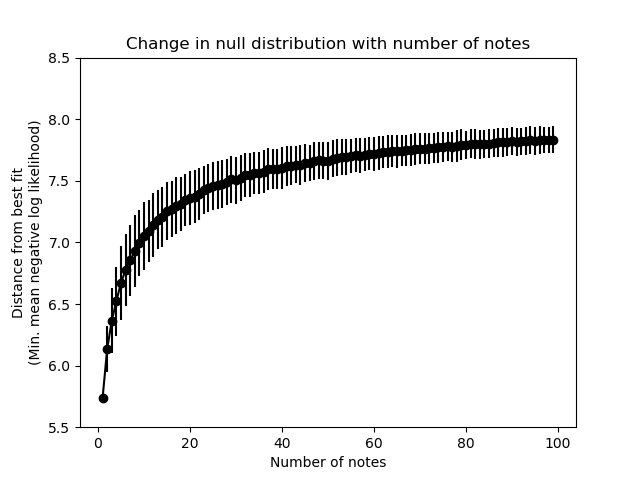
*

**Fig. S5. Visualization of key-finding performance with melody length.** The plot visualizes the change in mean (points) and standard deviation (error bars) of the null distribution of “best fit” returned by the PDF algorithm (major scale), where lower values indicate better fit. Plotted values here are based on 1,000 random-note permutations per melody length. Because the mean and standard deviation of random sequences changes with melody length, each improvisation must be z-scored against a large set of random-pitch sequences that match the number of notes and duration of each note.


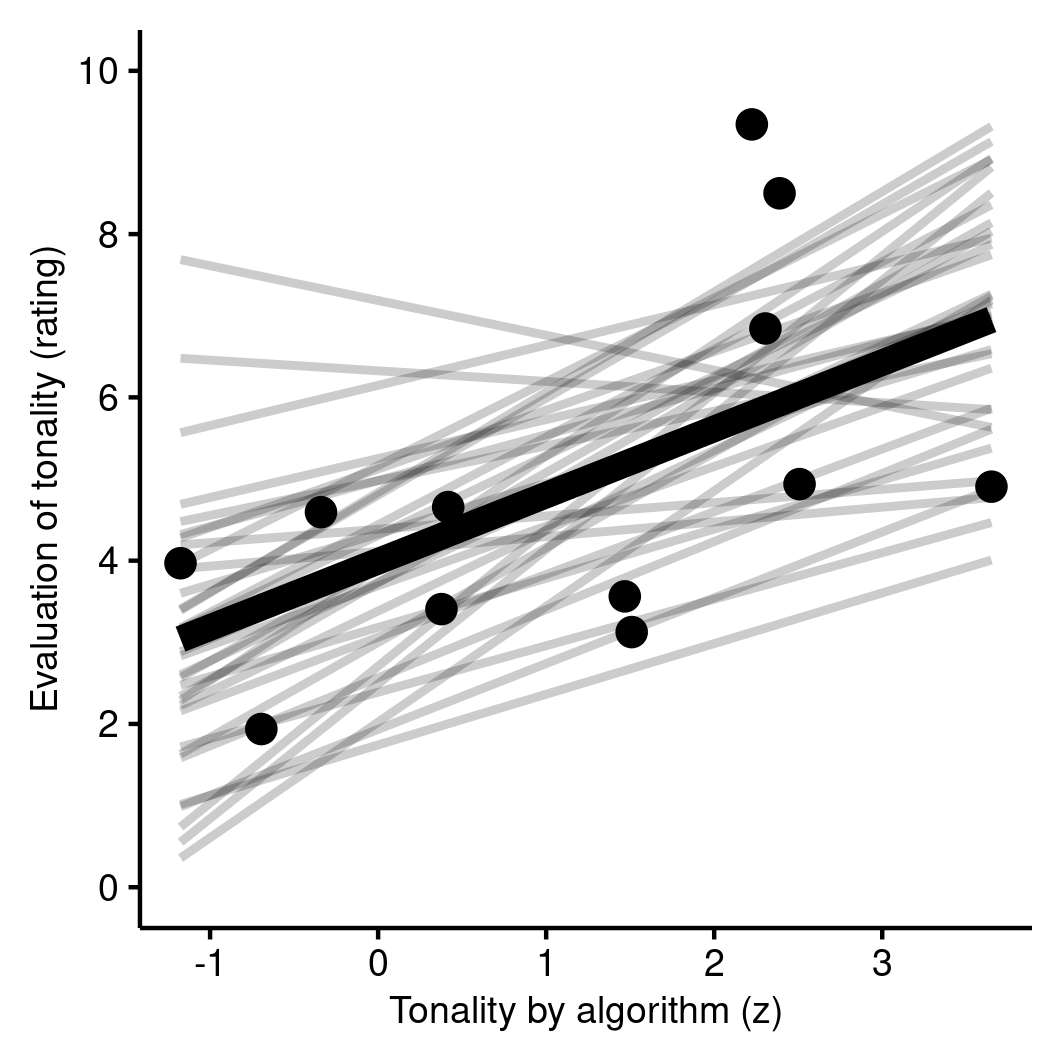


**Figure S6. Relationship between musicians’ evaluations of tonal coherence and z-score proportion of in-key notes.** The algorithmic approach taken in the analysis is expected to agree with listener experiences, but the degree to which they correlate is a matter of interest. We recruited 32 evaluators (20 female, 8 male, 4 did not indicate; *M* = 28.5, *SD* = 10.0 years, one value missing) with a minimum of 3 years of music theory (*M* = 6.4, *SD* = 4.6 years) or formal instrumental training (*M* = 10.0, *SD* = 4.4 years). Each rated 12 full improvisations (6 amusic, 5 control, 1 professional) from the free improvisation category (i.e., no musical or verbal prompt) which were selected to be similar in length (20–30 s) and number of notes (40–55 notes), but differing in z-scores of proportion of tonal notes (min. = -1.18, max. = 3.65). Audio was presented in an online task. Listeners were instructed not to perform a formal analysis, but rather, to evaluate their impression of the tonal coherence of the performance on a scale from 0 (‘not at all tonal’) to 10 (‘coherent with a single key’). The task included example melodies which were composed to be more coherent or less coherent with a single key. Average ratings of tonal coherence (*n* = 32 per each of 12 improvisations) correlated with z-scores, *r*(10) = .55, *p* = .032 (one-tailed), and are visualized as points and the bold line. On an individual level, the direction of the correlation coefficient was positive for 30 of 32 raters (binomial test *p* < .001; average coefficient = .41), visualized as light gray lines. In sum, despite the propensity to be generous for errors in vocal tuning^2^ and the unnaturalness of the evaluation task, listeners tended to agree with the results of the algorithm.


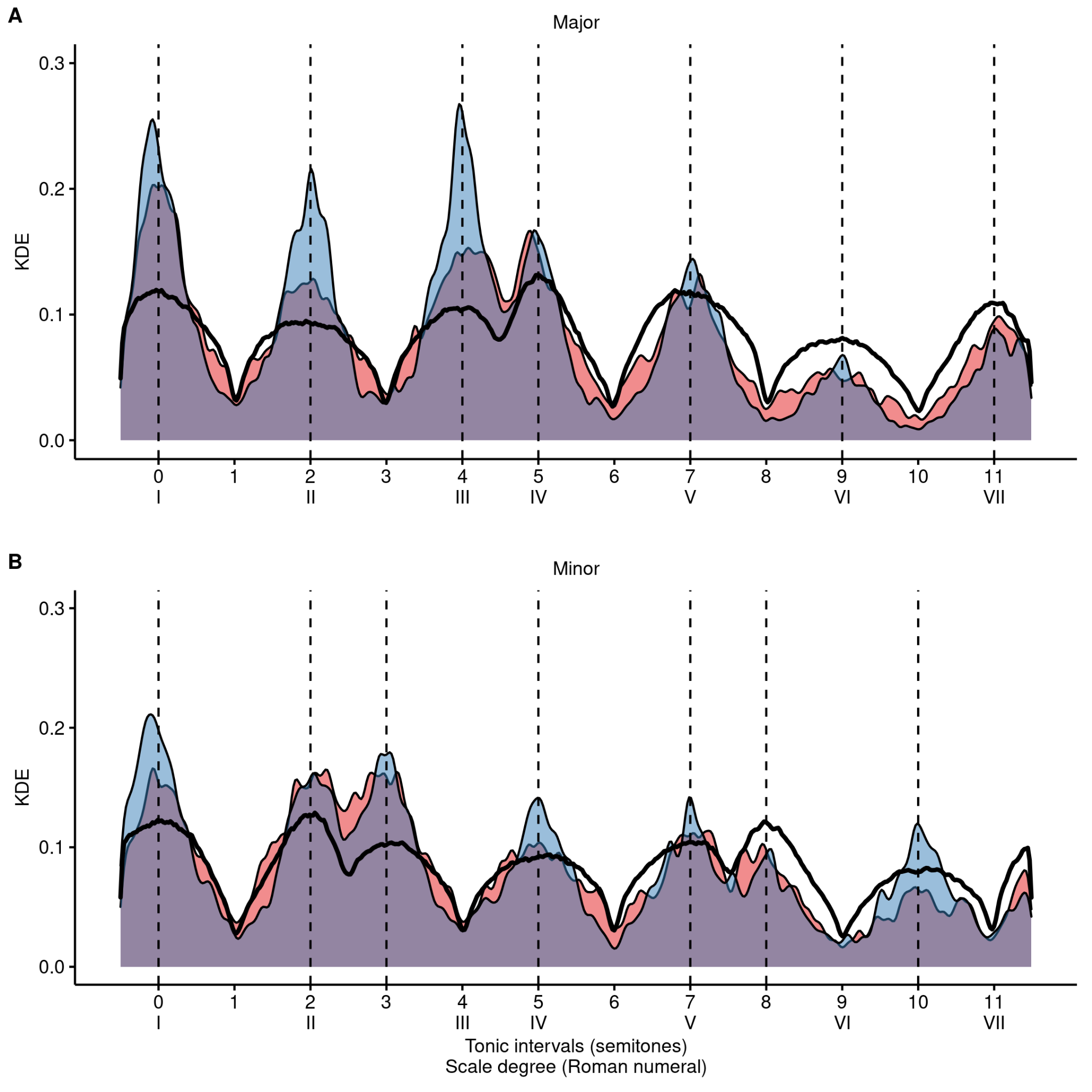


**Figure S7. Distribution of tonic intervals relative to null distribution.** The density plot of all notes sung during improvisations relative to the tonic, separately for major (Panel A) and minor (Panel B). Red indicates amusic > control, blue indicates control > amusic, and purple indicates overlap between groups. The more heavily weighted black line indicates a density plot representing a null distribution, derived from a sample of 1,000,000 random pitches used in the z-score analysis. Vertical dashed lines represent intervals in scale by mode. KDE = Kernel density estimate, an estimate of the probability density function; kernel bandwidth = 1/10.


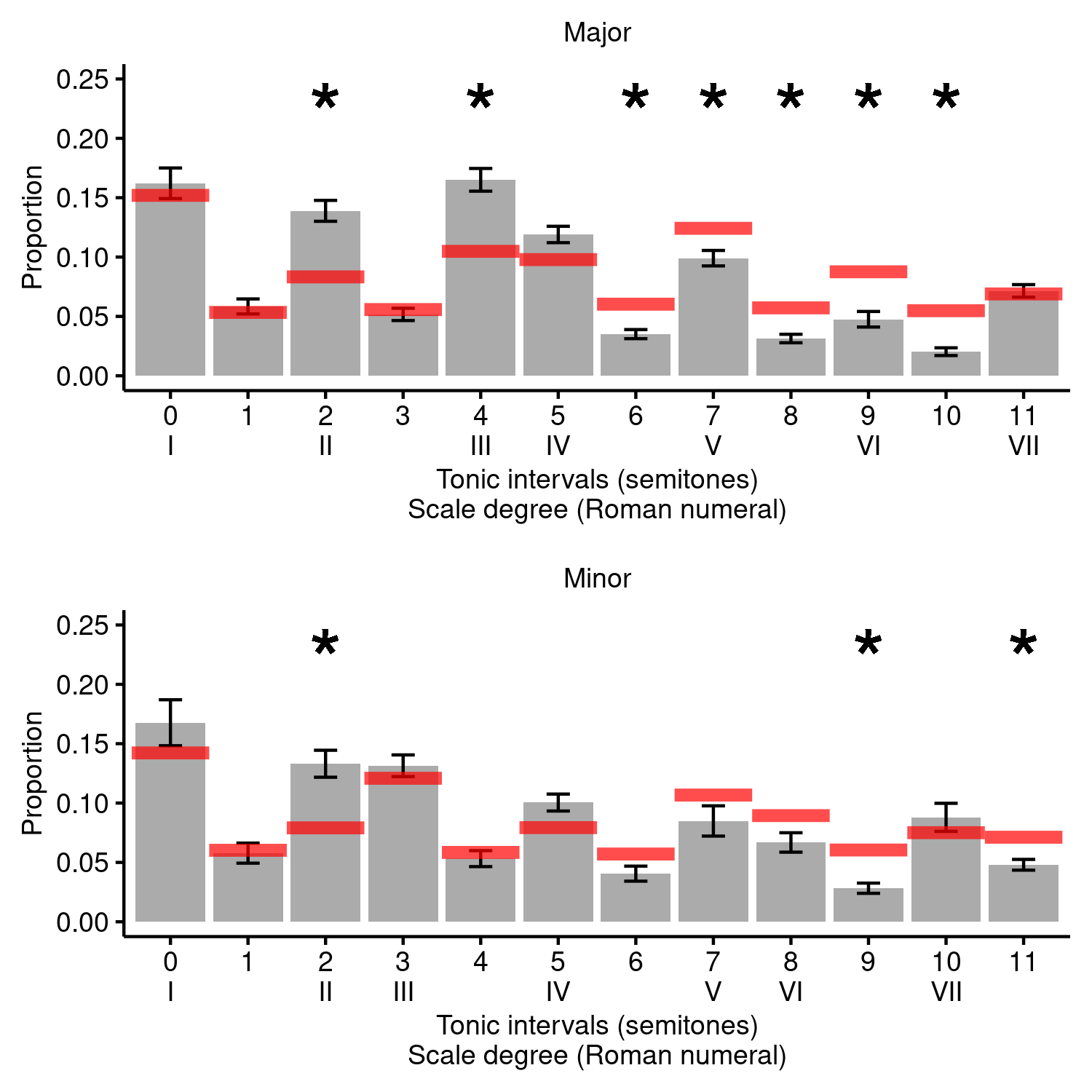


**Fig. S8. Proportion of notes as intervals relative to the tonic.** Tonal profiles generated from improvisations are visualized as grey bars. Only data from control participants and from trials without musical stems (n = 10 verbal prompts) are included here, to remove the possibility that observed deviations are due to perceptual deficits (amusia) or tonal induction (e.g., tonal or atonal stems). For comparison, probe tone ratings^3^ for the major (top) and minor scale (bottom) profiles are visualized as horizontal red lines. Asterisks represent a significant one-sample t-test for the proportion of notes (Bonferroni-Holm). Error bars are S.E.M. Music theory and the ratings predict a hierarchy of tonic (I) > fifth scale degree (V) > third scale degree (III) > out-of-scale notes. Here we observe some deviations from that pattern. In the major profile, scale degrees II and III were more prevalent than predicted by ratings, and higher scale degrees (V and VI) were less prevalent. Moreover, while the tonic (I) was more prevalent than predicted by ratings, it was not clearly more prevalent than scale degrees II or III. Improvisations in the minor mode generally followed the profile of ratings, except that the second scale degree (II) was again more prevalent than predicted by ratings.


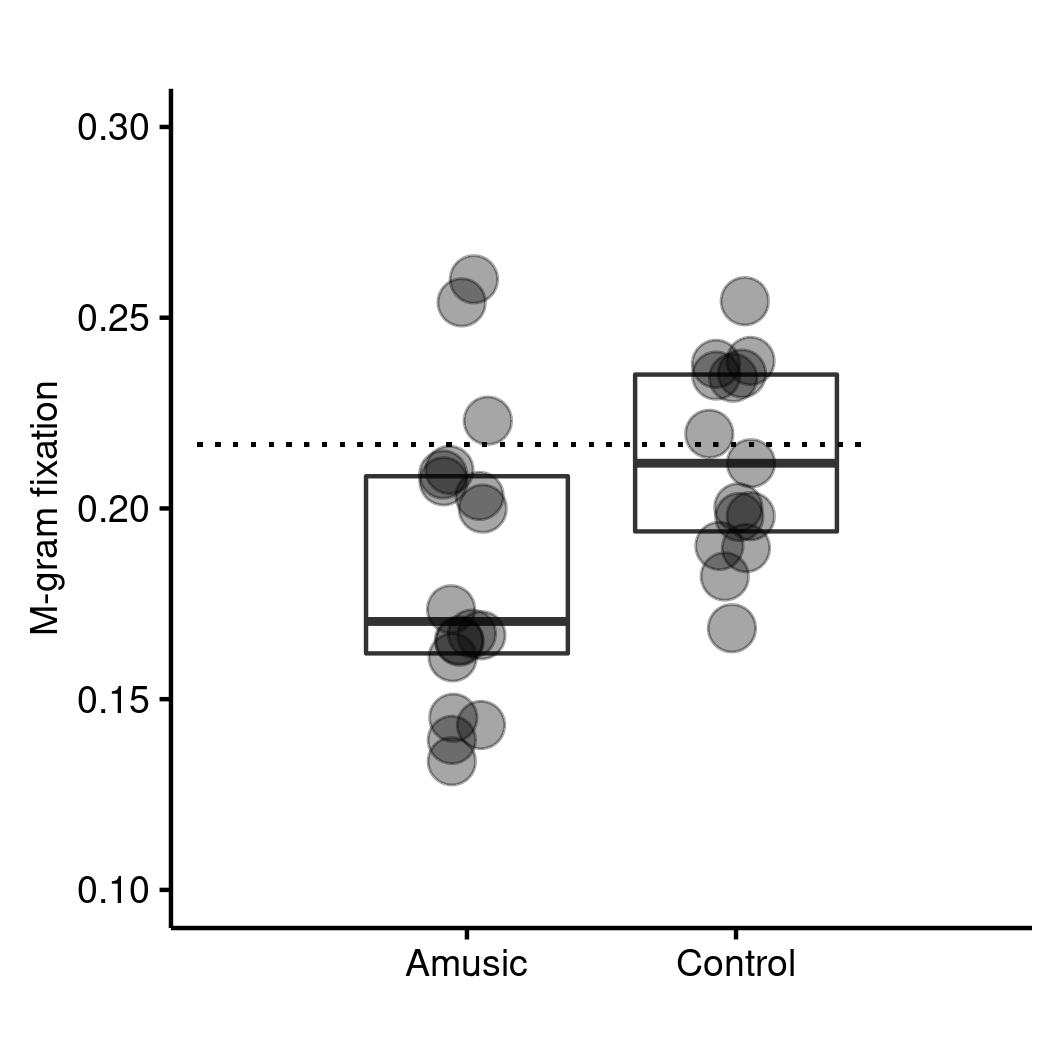


**Fig. S9.** **Analysis of repetition in improvisations.** An overreliance on repetition could boost scores of tonality artificially (e.g., singing the same note or pattern repeatedly). To rule out the possibility that participants with amusia were simply more reliant on repetition, we analyzed improvisations using “m-gram” analyses^4^. Following the convention of those analyses, we collapsed all possible intervals into 19 categories (-13 or less, -12, -11 or -10, -9 or -8, -7, -6, -5, -4 or -3, -2 or -1, 0 , +1 or +2, +3 or +4, +5, +6, +7, +8 or +9, +10 or +11, +12, +13 or more semitones). In order to maximize detection of repeating patterns of pitch, relative note duration was not considered. Pitch intervals of a melody were considered in windows of size *n* (i.e., number of intervals), sliding across the melody interval-by-interval. At each step, the intervals in that window, termed an *m*-token, were logged. Then, across all logged *m*-tokens, unique values were tallied, such that *m*-tokens occurring repeatedly were summed. If an observed *m*-token never repeats, that particular *m*-type sequence has a frequency of 1. The analysis was repeated by changing the size of the sliding window, *n*, for which we used values of 1–3 intervals (2–4 notes) to capture patterns of different lengths. For each window size *n*, the frequency of the *m*-type with the most repeats was divided by the total number of *m*-tokens at that window size. A summary score was calculated by averaging across window sizes. In other words, the more that an improviser repeats a single pattern, the closer the score will approach 1, and if they never repeat themselves, the score will be 0. The dashed line represents the scores of a professional singer.

The results suggest slightly more repetition in the improvisations of controls (*M* = .21, *SD* = .03) compared to those with amusia (*M* = .18, *SD* = .04), *t*(31) = 2.45, *p* = .020, but the group distributions overlapped considerably. The measure of repetition did not correlate with either measure of tonality: *z*-transformed proportion of tonal notes, *r*(31) = .19, *p* = .292; proportion of final note ending on the tonic, *r*(31) = .24, *p* = .177.


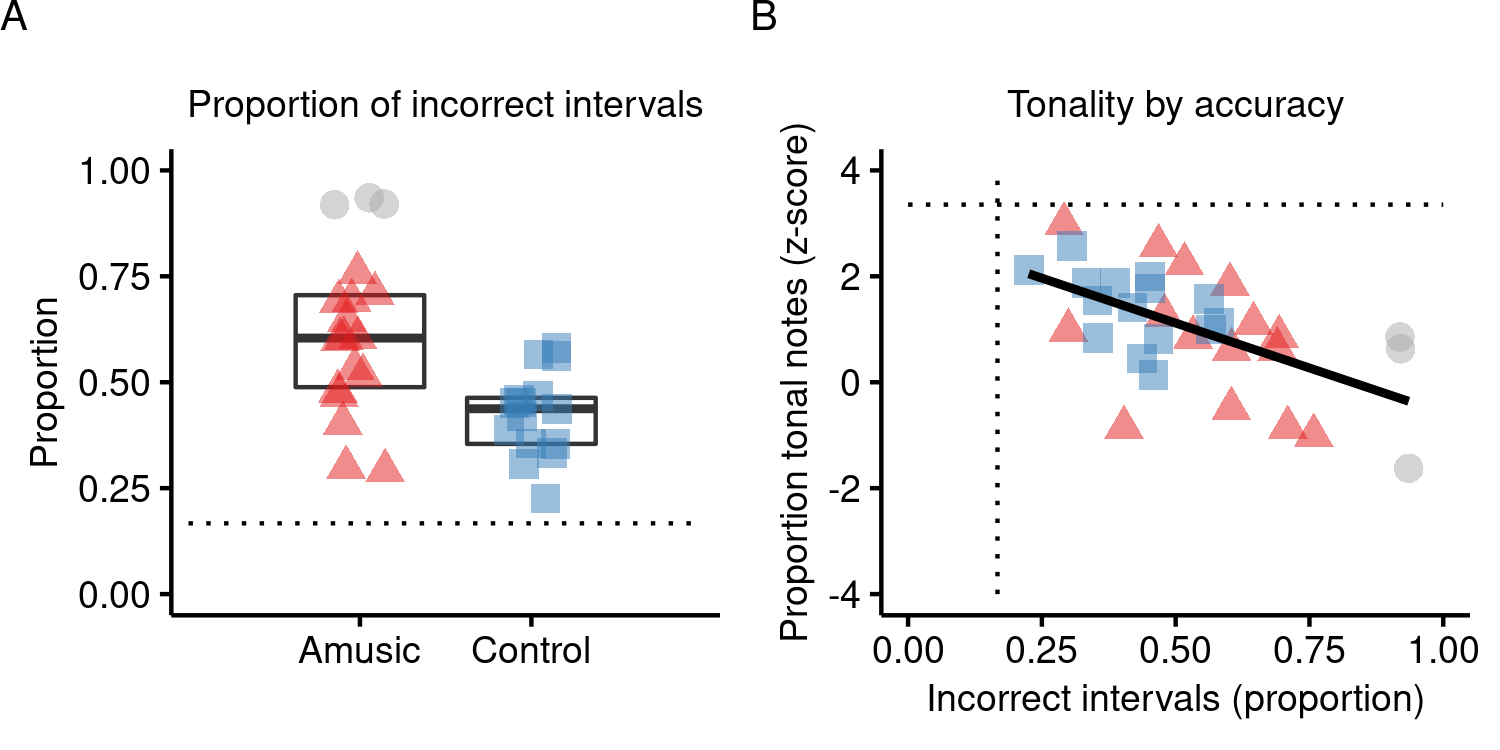


**Fig. S10. Singing accuracy for highly familiar tunes.** To provide an estimate of singing proficiency in each group and in the group with amusia in particular, we measured the proportion of incorrect intervals in the rendition of one highly familiar tune relative to the musical notation (Fig. S4). An interval was incorrect if it was not within 50 cents of the notated interval. Panel A shows the individual proportion of incorrect intervals. Controls (blue squares) show more accuracy than individuals with amusia (red triangles). The horizontal dashed line marks the score of a professional singer. As expected, participants with amusia performed more poorly than controls, *F*(1, 31) = 11.94, *p* = .002, *η_p_^2^* = .28. Note that 6 of 17 participants with amusia (~35%) in the present sample sang in tune or within 1 SD from the control group mean. Several individuals with amusia failed to generate a recognizable rendition of the melody without lyrics and improvised instead (gray circles), which could indicate inability to access the melody in memory or extremely poor singing. Altogether, the results replicate previous research showing that individuals with amusia sing familiar songs less accurately than controls^5–7^. We note that the proportion of incorrect intervals appears higher in the current data than in other research^5,6^, but we used stricter criteria, incorporating both direction and magnitude (i.e., less than 50 cents error rather than 100 cents). Even accurate untrained singers tend to produce a median interval deviation in the range of 50–100 cents^8^. Thus, the accuracy of the controls can be considered typical of untrained singers.

Panel B examines tonality of renditions of familiar tunes (z-transformed proportion of tonal notes) plotted against singing accuracy (proportion of incorrect intervals, averaged across lyric or no lyric attempts) by individual. If the errors are tonally consistent with the melody (e.g., substituting one in-scale note for another), then the proportion of tonal notes should be high regardless of the number of errors. Alternatively, if singing errors deviate from the scale, then the tonality of the performance should decrease when the amount of singing error increases. Separate one-sample *t*-tests showed that the tonality of familiar renditions was above chance (z-score of 0) for the controls as a group, *M* = 1.41, *SD* = 0.69, *t*(14) = 8.15, *p* < .001, *d* = 2.10, 95% CI [1.04, 1.78], as well as for the group with amusia, *M* = 0.70, *SD* = 1.27, *t*(17) = 2.34, *p* = .032, *d* = .55, 95% CI [0.07, 1.34]. The tonality was greater than chance in absolute terms (i.e., z-score of 0) for 13 of 18 amusics and 15 of 15 controls. However, there was a negative correlation between the proportion of incorrect intervals and the z-transformed proportion of tonal notes in familiar tune performances, *r*(31) = -.57, *p* < .001, 95% CI [-.76, -.28] (solid trend line), indicating that more errors in accuracy coincide with more deviations from tonality. The dashed vertical and horizontal lines mark the scores of a professional singer.

Singing inaccuracy on familiar tunes also correlated negatively with both measures of tonality for improvisations: z-transformed proportion of tonal notes, *r*(31) = -.56, *p* < .001, 95% CI [-.76, -.27]; proportion of final notes ending on the tonic, *r*(31) = -.53, *p* = .001, 95% CI [-.74, -.23]. In short, inaccurate singers tended to be less tonal improvisers.

**Movie S1 (separate file).** **Movie file visualizing key-finding algorithm.** The upper plot visualizes the continuous probability density function (PDF) of the Krumhansl-Kessler^3^ ratings for the minor mode. Size and position of the red dots represent the position and height of the histogram bars in the middle plot, respectively. The histogram bars in the middle plot represent the distribution of pitches across the octave, with height corresponding to pitch duration. The rightward shift represents transposition of the distribution in small steps. Note that bins/steps of 10 cents are used here for visualization, whereas the algorithm uses bins/steps of 1 cent. The lower plot represents the weighted mean of the negative log likelihood at each step, i.e., mean of NLL at each red dot index in the upper panel weighted by dot size. Lower values represent better fit, and a minimum value is reached at index 729 (i.e., transposition of 729 cents), which can be used to derive the tonic. The static red bars that appear when the minimum is reached represent the “best fit” between the PDF in the upper panel and the histogram in the middle panel.

**Audio S1 (separate file). Audio file of a more tonal improvisation.** The song was recorded by a participant with amusia to the verbal prompt, ‘love song’. This item received a z-score of 1.74, corresponding to 22 of 26 notes (84.6%) being in the determined scale, and ending on the tonic. The key-finding analysis of this file is visualized in Figure 1.

**Audio S2 (separate file).** **Audio file of a less tonal improvisation.** The song was recorded by a participant with amusia to a short musical stem (not provided). This item received a z-score of -2.18, corresponding to 14 of 24 notes (58.3%) being in the determined scale, and with the final note *not* matching the determined tonic.

**Audio S3 (separate file).** **Audio file of a more tonal improvisation.** The song was recorded by a nonmusician control participant to a short musical stem (not provided). This item received a z-score of 2.52, corresponding to 19 of 20 notes (95.0%) being in the determined scale, and with the final note matching the determined tonic.

**Audio S4 (separate file).** **Audio file of a less tonal improvisation.** The song was recorded by a nonmusician control participant to the prompt “sad song”. This item received a z-score of -1.71, corresponding to 15 of 25 notes (60.0%) being in the determined scale, and with the final note *not* matching the determined tonic.

**SI References**

1. Peretz, I., Champod, A. S. & Hyde, K. Varieties of musical disorders. *Ann. N. Y. Acad. Sci.* **999**, 58–75 (2003).

2. Hutchins, S., Roquet, C. & Peretz, I. The vocal generosity effect: How bad can your singing be? *Music Percept.* **30**, 147–159 (2012).

3. Krumhansl, C. L. & Kessler, E. J. Tracing the dynamic changes in perceived tonal organization in a spatial representation of musical keys. *Psychol. Rev.* **89**, 334–368 (1982).

4. Müllensiefen, D. Fantastic: Feature ANalysis Technology Accessing STatistics (In a Corpus): Technical Report v1.5. 37 (2009).

5. Dalla Bella, S., Deutsch, D., Giguère, J.-F., Peretz, I. & Deutsch, D. Singing proficiency in the general population. *J. Acoust. Soc. Am.* **121**, 1182–1189 (2007).

6. Dalla Bella, S., Giguère, J.-F. & Peretz, I. Singing in congenital amusia. *J. Acoust. Soc. Am.* **126**, 414–424 (2009).

7. Tremblay-Champoux, A., Dalla Bella, S., Phillips-Silver, J., Lebrun, M.-A. & Peretz, I. Singing proficiency in congenital amusia: Imitation helps. *Cogn. Neuropsychol.* **27**, 463–476 (2010).

8. Pfordresher, P. Q. & Brown, S. Vocal mistuning reveals the origin of musical scales. *J. Cogn. Psychol.* **29**, 35–52 (2017).
